# Development and Feasibility of a Digital Acceptance and Commitment Therapy–Based Intervention for Generalized Anxiety Disorder: Pilot Acceptability Study

**DOI:** 10.2196/21737

**Published:** 2021-02-09

**Authors:** Nicola R Hemmings, Jamie M Kawadler, Rachel Whatmough, Sonia Ponzo, Alessio Rossi, Davide Morelli, Geoffrey Bird, David Plans

**Affiliations:** 1 Department of Organizational Psychology Birkbeck University of London London United Kingdom; 2 BioBeats Group Ltd London United Kingdom; 3 Work With Wellbeing London United Kingdom; 4 Salomons Institute for Applied Psychology Canterbury Christ Church University Kent United Kingdom; 5 Department of Computer Science University of Pisa Pisa Italy; 6 Department of Engineering Science Institute of Biomedical Engineering University of Oxford Oxford United Kingdom; 7 Department of Experimental Psychology University of Oxford Oxford United Kingdom; 8 Social, Genetic and Developmental Psychiatry Centre Institute of Psychiatry, Psychology and Neuroscience King’s College London London United Kingdom; 9 Initiative in the Digital Economy, Department of Science, Innovation, Technology, and Entrepreneurship University of Exeter Exeter United Kingdom

**Keywords:** anxiety, depression, acceptance and commitment therapy, person-based approach, mHealth, mental health, digital, remote, smartphone, mobile phone

## Abstract

**Background:**

Generalized anxiety disorder (GAD) is characterized by excessive worry that is difficult to control and has high comorbidity with mood disorders including depression. Individuals experience long wait times for diagnosis and often face accessibility barriers to treatment. There is a need for a digital solution that is accessible and acceptable to those with GAD.

**Objective:**

This paper aims to describe the development of a digital intervention prototype of acceptance and commitment therapy (ACT) for GAD that sits within an existing well-being app platform, BioBase. A pilot feasibility study evaluating acceptability and usability is conducted in a sample of adults with a diagnosis of GAD, self-referred to the study.

**Methods:**

Phase 1 applied the person-based approach (creation of guiding principles, intervention design objectives, and the key intervention features). In Phase 2 participants received the app-based therapeutic and paired wearable for 2 weeks. Self-report questionnaires were obtained at baseline and posttreatment. The primary outcome was psychological flexibility (Acceptance and Action Questionnaire-II [AAQ-II]) as this is the aim of ACT. Mental well-being (Warwick-Edinburgh Mental Well-being Scale [WEMWBS]) and symptoms of anxiety (7-item Generalized Anxiety Disorder Assessment [GAD-7]) and depression (9-item Patient Health Questionnaire [PHQ-9]) were also assessed. Posttreatment usability was assessed via self-report measures (System Usability Scale [SUS]) in addition to interviews that further explored feasibility of the digital intervention in this sample.

**Results:**

The app-based therapeutic was well received. Of 13 participants, 10 (77%) completed the treatment. Results show a high usability rating (83.5). Participants found the digital intervention to be relevant, useful, and helpful in managing their anxiety. Participants had lower anxiety (*d*=0.69) and depression (*d*=0.84) scores at exit, and these differences were significantly different from baseline (*P*=.03 and .008 for GAD-7 and PHQ-9, respectively). Participants had higher psychological flexibility and well-being scores at exit, although these were not significantly different from baseline (*P*=.11 and .55 for AAQ-II and WEMWBS, respectively).

**Conclusions:**

This ACT prototype within BioBase is an acceptable and feasible digital intervention in reducing symptoms of anxiety and depression. This study suggests that this intervention warrants a larger feasibility study in adults with GAD.

## Introduction

### Background

Generalized anxiety disorder (GAD) is diagnosed if an individual has excessive worry that is difficult to control for more days than not over a period of 6 months [1]. GAD is associated with an increased reactivity to, and avoidance of, internal experiences [2]. There is high comorbidity of GAD with other anxiety (51.7%) and mood (63%) disorders [3], especially with depression (*r*=.62) [4].

Just under half of individuals with GAD suffer for 2 years before correctly being diagnosed [5], and even after diagnosis, waiting times for treatment are up to 18 weeks [6].

A digital therapeutic tool has the potential to increase accessibility and availability of treatment for those suffering with GAD by reducing barriers such as waiting times before treatment [7], perceived stigma [8,9], geographical distance, financial costs, and lack of time due to, for example, work commitments or caring responsibilities [10]. Digital interventions, whether therapist-guided or self-guided, have been shown to be as effective as face-to-face interventions [11,12] when based on the same core processes of face-to-face treatment with only the mode of delivery changing [13].

### Acceptance and Commitment Therapy as a Treatment for GAD

Acceptance and commitment therapy (ACT) is a form of cognitive behavioral therapy, rooted in functional contextualism [14,15] and relational frame theory [16].

ACT aims to increase psychological flexibility, or the ability to deal with challenging experiences in a flexible way while continuing to act based on one’s values [17]. Psychological flexibility reduces experiential avoidance and the unwillingness to experience difficult emotions, thoughts, or sensations [17].

The aforesaid reduction of experiential avoidance and increase in psychological flexibility are achieved via 6 core processes of change that are closely interlinked. *Contact with the present moment* is the nonjudgmental present moment awareness of one’s internal and external environment. *Self-as-context* is noticing that one is not just ones’ thoughts, emotions, and self-image, but they are also the observer of them. *Cognitive defusion* includes techniques aiming to treat thoughts and feelings for what they are (mental imagery, streams of words, sensations), not as truths that must be reacted to or get caught up in. *Acceptance* means allowing unpleasant feelings, rather than trying to change them. *Values* are knowing what matters in life to provide meaning and direction. *Committed action* is taking targeting value-based action and doing what it takes, even when difficult [17-19].

Psychological flexibility is proposed to be a fundamental aspect of health and well-being [20]; even though ACT does not aim to specifically eliminate symptoms of anxiety, the ACT model has a transdiagnostic approach and has been consistently shown to reduce symptoms of anxiety [21] and depression [22] among other mood disorders via its 6 core processes. This effect could be due to the reduction of “experiential avoidance,” a proposed concept of anxiety [2]. Experiential avoidance is the attempt to avoid or control internal experiences, rather than accepting them. This fear of losing control over one’s emotional responses (in particular anxiety) is seen as the opposite of being psychologically flexible; therefore, increasing psychological flexibility can reduce experiential avoidance, and thus increase psychological flexibility.

ACT has been used in digital interventions and has shown to be an efficacious and acceptable treatment for adults with anxiety disorders [23], and there is early support for therapist-guided and self-guided digital ACT-based effectiveness reducing anxiety in populations with anxiety disorders [24-26] and the general population [27-29].

However, poor engagement rates are a well-documented and an ongoing concern in the design, development, and evaluation of digital interventions [30-34]. Self-guided interventions suffer from even greater dropout than therapist-guided interventions [35], potentially due to the increased support that these can offer [36]. However, reasons for poor engagement and dropout are not consistently reported in the literature; a systematic review from 2010 reported a wide range of adherence rates (2%-83% [37]) in 16 studies using internet-based interventions and specifically called for analysis of variables associated with dropout. Consistent reporting of these variables remains an issue in the literature [38-40]. To increase engagement, which is linked to efficacy [41], more research is needed to determine whether adopting an accepted framework, such as the person-based approach (PBA), could serve as a potential first step in designing interventions with greater engagement rates.

### Person-Based Approach

The PBA is a systematic framework for designing interventions that addresses and accommodates perspectives of the people who will use them. Adopting a PBA addresses the ongoing concern of a lack of engagement in digital interventions [42,43], with the aim of increasing the likelihood of achieving the desired therapeutic outcome. It goes beyond the traditional system of collecting user feedback as it addresses a person’s experience of the intended behavior change techniques [43].

The approach is conducted over 2 stages to create a persuasive, feasible, and relevant digital intervention [43]. First, the development stage creates a *persona*, a summary representation of qualitative research conducted with a wide range of people from the target user’s population, showcasing a deep understanding of their psychosocial context alongside their views on the proposed intervention. The second stage identifies *guiding principles* that inform the intervention development in addressing the persona’s key context-specific behavioral issues [43]. Understanding the user’s views and psychosocial context enables the ability to address barriers to engagement and therefore, increase acceptability, feasibility, and efficacy of the digital intervention [44].

The PBA is a useful methodology in designing digital interventions; however, to our knowledge, there has been no published research on using the PBA for the development of a suitable self-guided digital intervention for GAD.

### Aim

This paper describes the development of a digital intervention via a mobile app for GAD, based on ACT using the PBA. Furthermore, it reports the results of a pilot study evaluating the prototype digital intervention in a sample of adults with self-referred GAD. The pilot study is designed to (1) evaluate the acceptability and usability of ACT-based content in a digital format derived using the PBA, and (2) provide a possible trend of the efficacy of this intervention with respect to psychological flexibility, mental well-being, and symptoms of anxiety and depression.

## Methods

### Phase 1: Development

#### Technical Platform

The technical platform used in this study was a pre-existing multidimensional well-being app, BioBase (BioBeats Ltd). BioBase is a smartphone app that contains several features that cover the core processes of ACT (aware, open, and active).

The ACT-based modules that provided psychoeducational information and activities on the core processes were shown via an in-app course named “Find Your Way.” The course was complemented by in-app BioBase tools (screenshots can be found in Multimedia Appendix 1).

To support the ACT pillars of “Aware,” “Open,” and “Active,” mood tracking is available through an Ecological Momentary Assessment (EMA; [45]), which allows individuals to increase their awareness of their emotional state by choosing a mood from a list of options and specify any ecological component surrounding the moment they chose to declare their mood (ie, where they were, whether they were alone or with somebody, and what activities they were engaged in). EMAs have been shown to be a valuable mood-tracking tool in the context of digital therapeutics aiming to reduce levels of anxiety and depression [46].

In addition to the mood tracking tool, an in-the-moment breathing exercise for stress reduction and a mindfulness-based progressive relaxation tool (Body Scan) were used to reduce symptoms of anxiety and depression through either heart rate variability biofeedback [47,48] or awareness of body sensations [49].

Finally, passive data collection on physical activity (ie, the number of steps performed every 20 seconds) and sleep continuity (eg, hours slept, number of awakenings) was achieved via the paired wearable device, BioBeam, and data on sleep and physical activity history were displayed on in-app dashboards. Awareness of and insight into one’s own sleep and activity patterns have been shown to affect anxiety and well-being [50,51].

All tools provided in-app feedback and recommendations to encourage positive behavior change.

The BioBase app, previously containing content for workplace stressors, has been shown to increase well-being and decrease anxiety after 4 weeks of use [52,53].

#### Intervention

##### Stage 1: Development of a GAD Persona

A persona defines the guiding principles of the intervention: a collection of symptoms, desired health behaviors, barriers to treatment, and desired impact of the intervention. Such a persona was created to understand the current psychosocial context of the target population (individuals with GAD).

The Diagnostic and Statistical Manual of Mental Disorders, 5th edition (DSM-5) [50] was consulted to clarify the current diagnostic criteria. Clinically recommended therapies, and their critiques, were investigated alongside models of GAD, self-help strategies, and confounding factors to their effectiveness [19,21,23,54-57]. This review of the literature highlighted behavioral issues specific to GAD, centered around explicit or voluntary cognitive avoidance strategies, and avoidance of internal experiences.

To create relevant scenarios in which to base an intervention, a persona narrative was created by a psychologist (NH) which was centered around everyday life for a person with GAD. These narratives were based on the psychologist’s experience of user feedback from previous BioBase studies with individuals with high anxiety [52,53] in conjunction with the aforementioned review of the literature. Examples include:

I enjoy my job but struggle to concentrate and feel guilty for taking time off when my anxiety is overwhelming

I just feel like I am always on edge and it is affecting my work and relationships

I don’t know what to do, I am just always anxious and can’t stop it

I am always tired and can never concentrate

I feel like there is this looming dread - but I'm not sure where it is coming from

My whole body is exhausted, I feel constantly tense, all my muscles ache

I can’t remember when I last slept well

In addition to the narratives, clear user journeys were created highlighting how a person would receive and interact with the digital intervention. A list of desired outcome behaviors was also collated to inform the intervention design.

##### Stage 2: Intervention Design and Creation

A prototype digital intervention was constructed that targeted the desired outcome behaviors and integrated into the existing National Institute for Health and Care Excellence’s (NICE) stepped treatment pathways for GAD [58]. The NICE stepped treatment pathway organizes the provision of service based on the severity of an individual’s symptoms. Initial steps involve monitoring and provision of relevant information. This escalates to self-help or therapist-guided interventions and then to medication and combined interventions [59].

The *intervention design objectives* were to reduce symptoms of GAD (ie, excessive worry and anxiety, linked to increased reactivity to and avoidance of internal experiences) by means of increasing psychological flexibility.

The *key features* that could address these aims are provision of education and guidance on anxiety, specific therapeutics to help the users to be open to and aware of their internal experiences, guidance on defusing and accepting difficult internal sensations, and advice as to how to clarify personal values and reduce barriers to life goals.

The content was curated by a psychologist experienced in creating engaging digital interventions (NH), based on the targeting of key behaviors, efficacy of ACT processes [60], exercises in a digital format [27,61], and use of visual metaphors for engagement with the program.

The treatment aimed to increase the concept of psychological flexibility through psychoeducation and exercises based on the 3 pillars of ACT: Aware, Open, and Action. Across these 3 pillars, the treatment program is structured around the 6 core processes of ACT: present moment, self-as-context, cognitive defusion, acceptance, values, and committed action (Table 1). This feasibility study investigated a prototype of an initial 6 modules (of 30 total modules designed)—1 module for each core ACT process. Each module contained the same structure: a brief overview of a metaphor that describes a core ACT process, a description of the metaphor (that can be accessed via text or audio file), and an activity to complete with a free text box in which the users can apply the therapeutic concept to their current scenario.

The 6 modules, each taking less than 5 minutes to read/listen, could be accessed and completed in any order, as previous research has shown no consistent user preference for guided or unstructured user journey [62]. The user was free to spend as long as necessary on the activities as in other digital interventions [28,62]. A schedule of 1 module per day was recommended to participants. Each module contained a follow-up notification on completion, which reminded the users to be cognizant of the concept that they had worked through.

**Table 1:** The structure of the digital intervention prototype. The outline evidences the 3 core pillars of ACT^a^ alongside the 6 ACT processes and links the intervention design objectives with the intervention key features (module name and exercise).

Module number	Pillar	Process	Intervention design objective	Key feature (Exercise)	Module name
1	Aware	Present moment	The users have increased awareness of what is driving their decisions in life (thoughts, feelings, emotions, and urges).	Passengers on the bus. Notice what thoughts, emotions, feelings, and sensations you are carrying with you.	In your driving seat
2	Aware	Self-as-context	The users have a greater awareness of their internal narrative directed by their thoughts and its effect on their behavior and decisions throughout the day. They check that they are basing decisions and behavior on reality, not the story their thoughts are telling them.	Noticing if the story your thoughts are telling you is different from reality.	The storyteller
3	Open	Cognitive defusion	The users are better able to notice and distinguish between thoughts that are either helping them or preventing them from reaching their goals and then build a positive mindset by attending to helpful thoughts.	Labeling thoughts as helpful or unhelpful.	Is this helpful?
4	Open	Acceptance	The users are aware of difficult emotions, thoughts, or urges they are experiencing, and the short-term gains and long-term costs of their avoidance behaviors.	Journaling the short- and long-term impact of current avoidance behaviors.	What are you avoiding?
5	Active	Values	The users connect with their values and what really matters to them to live a meaningful, valued life.	Journaling what a loved one says about you during an anniversary speech.	Your attention, please
6	Active	Committed Action	The users state that they are willing to experience initial discomfort in order to achieve meaningful goals for them.	Acknowledging barriers and creating an action plan.	ACTion plan

^a^ACT: acceptance and commitment therapy.

To safely address readability and clarity of the modules, iterative testing was conducted with 10 paid user testers with no pre-existing anxiety. Modifications were made to simplify the language and to improve the clarity of what is addressed by the activity. The app was then further reviewed by a clinical psychologist (RW), who has experience using ACT-based approaches in GAD, whose feedback was fed into the program design via the PBA framework.

To meet the specific behavioral context of GAD (such as excessive worry) the overall design of the app was intended to be relaxing and calming. The home screen changes color based on the time of day to anchor the persons to their present environment. The tone and style of the language used were accessible, nonjudgmental, friendly, and supportive and avoided explicitly mentioning any specific medical conditions or diagnosis (unless necessary within therapeutic Step 1: “Information on anxiety”). All modules had a Flesch Kincaid reading ease score [63] of 71.5 or above suggesting it should be easily understood by individuals aged 13 and above.

#### Phase 2: Feasibility Testing

##### Overview

A feasibility test of the initial prototype app–based therapeutic (testing acceptability, usability, and efficacy) was conducted in a sample of individuals who have reported a diagnosis of GAD from a mental health professional as the first step in an iterative cycle of development and evaluation. The study took place in February-March 2020.

##### Participants

Participants were recruited from a pool of subjects excluded from a previous study in student mental health [52], because of a declaration of diagnosed mental health disorder. Based on similar feasibility studies [25], up to 20 participants were contacted. Participants were paid £20 (~US $27) for their time to take part.

Inclusion criteria were age over 18, self-reported clinical diagnosis of GAD from a general practitioner (GP) or mental health professional, able to read and understand English, and access to an iPhone (6 or above). All participants were given a participant information sheet and provided consent via the consent form. Ethical approval was obtained from the University of Exeter Research Ethics Committee (eUEBS003011).

##### Procedure

At baseline, demographic information and the baseline questionnaire data were collected. Participants were then given a unique activation code and sent a video and study information pack to provide a clear overview of what they would be required to do (interact with the app every day for at least 5 minutes a day, using whichever tools they feel comfortable with, and complete all 6 ACT modules in the 2-week period), as well as relevant contacts for research-related questions and mental health emergencies. At 2 weeks after app download, exit questionnaire data were collected and access to the app ceased.

Treatment dosage was defined as completion of all 6 ACT modules and participants were excluded if they did not complete all modules. Participants were given the option to take part in semistructured interviews with an experienced psychologist (JK) after the exit questionnaire was completed.

##### Duty of Care

As this population is at risk of severe mental health issues, a duty of care protocol was implemented if the research team felt there was a significant concern for the participant’s welfare. In the demographics section, each participant was required to input details of an emergency contact and his/her GP; participants were excluded from the study if they failed to provide this information. If on either the baseline or exit questionnaire participants stated they had thoughts of self-harm or suicide or both over the past 2 weeks, the research team would contact the at-risk participant via email with relevant helpline information, the participant’s emergency contact by email with relevant information on how to support someone during a mental health crisis, and also the participant’s GP via a letter with information on the study and questionnaire results.

##### Outcome Measures

Psychological flexibility, or the ability to be aware of and deal with difficult emotions while acting in accordance with one’s values, was measured by the 7-item Acceptance and Action Questionnaire-II (AAQ-II), which utilizes a 7-point Likert response scale (scores range 10-70 with lower scores indicating greater psychological flexibility) [64].

Mental well-being was assessed using the 14-item Warwick-Edinburgh Mental Well-being Scale (WEMWBS), which utilizes a 5-point Likert scale for responses (scores range from 14 to 70, with higher scores indicating greater mental well-being) [65].

The 7-item Generalized Anxiety Disorder Assessment (GAD-7) [66] and the 9-item Patient Health Questionnaire (PHQ-9) [67] are clinical assessment tools for measuring anxiety and depression, respectively, over the previous 2 weeks. GAD-7 scores range from 0 to 21 and PHQ-9 scores from 0 to 27; both have a cutoff of 10, indicating moderate symptoms which warrant referral to a mental health professional.

Feedback questions were asked as part of the exit questionnaire, regarding learned concepts, any behavior change, and any benefits received as a result of the intervention. The System Usability Scale (SUS) was included as part of the exit questionnaire to assess usability of the app; it assesses the ability of the participant to effectively and efficiently complete tasks using the system, as well as the participant’s satisfaction in using the app. The scale provides a single number and corresponding grade [68].

Optional semistructured interviews were conducted to further explore feasibility of the intervention in the participant’s own words, in terms of usability of the app and acceptability of the ACT-based content (ie, if the participants felt the content was relevant to the anxiety they struggle with). Questions were based around downloading the app and setting up the paired wearable, accessing the modules, experience using the tools and features (ie, usability), and experience during and after completion of the modules and if any aspect of the content triggered anxiety (ie, acceptability).

#### Statistical Analysis

To explore Aim 1 (evaluate the acceptability and usability), the SUS was reported, and a thematic analysis of the feedback questionnaires and semistructured interviews were conducted.

To explore Aim 2 (exploratory analysis to evaluate the preliminary efficacy of this intervention), differences between baseline and exit scores of the AAQ-II, WEMWBS, GAD-7, and PHQ-9 were analyzed. Because of small sample size, nonparametric paired-sample Wilcoxon signed-rank tests were carried out using R [69]. Differences were considered significant if *P*<.05. Effect sizes were calculated as Z/√N and interpreted in accordance with Cohen’s classification of effect sizes (ie, 0.2 [small effect], 0.5 [moderate effect], 0.8 [large effect]) [70].

## Results

### Participants

**Figure 1:**
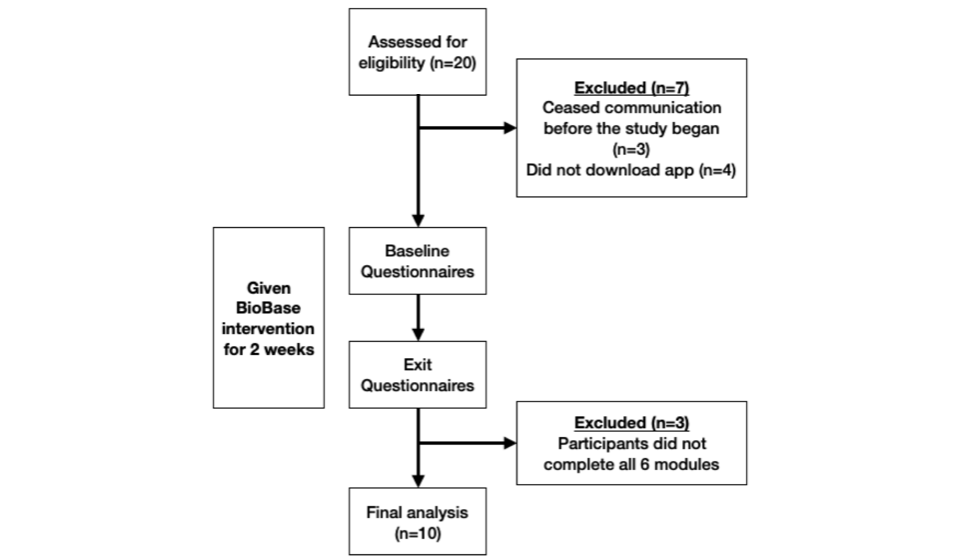
Study flow chart.

A total of 13 participants met inclusion criteria, were eligible to take part in the study, and agreed to do so. Seven participants failed to meet inclusion criteria, as they ceased communication before the study could begin (n=3) or did not download the app (n=4). Three participants were excluded from the analysis because they did not complete all 6 modules (1 participant completed no modules and 2 participants completed 1 module). The final sample consisted of 10 participants; of these 10, 7 participated in the semistructured interview after the exit questionnaire. CONSORT flow chart is presented in Figure 1. Demographics are presented in Table 2.

**Table 2:** Baseline characteristics of the study sample (N=10).

Variable		Value
Age (years), mean (range)		25.2 (19-48)
**Gender, n**		
	Female	10
	Male	0
**Education, n (%)**		
	School to age 16	2 (20)
	College/A-levels to age 18	7 (70)
	Undergraduate degree	1 (10)
**Undertaking therapy/counseling, n (%)**		
	Yes	2 (20)
	No	8 (80)
**On medication for anxiety, n (%)**		
	Yes	8 (80)
	No	2 (20)

### Duty of Care

Seven participants responded to item 9 of the baseline or exit PHQ-9, indicating they had thoughts of self-harm/suicide in the previous 2 weeks; the participant, the participant’s emergency contact, and the participant’s GP were then contacted as per the duty of care protocol.

### Engagement

**Figure 2:**
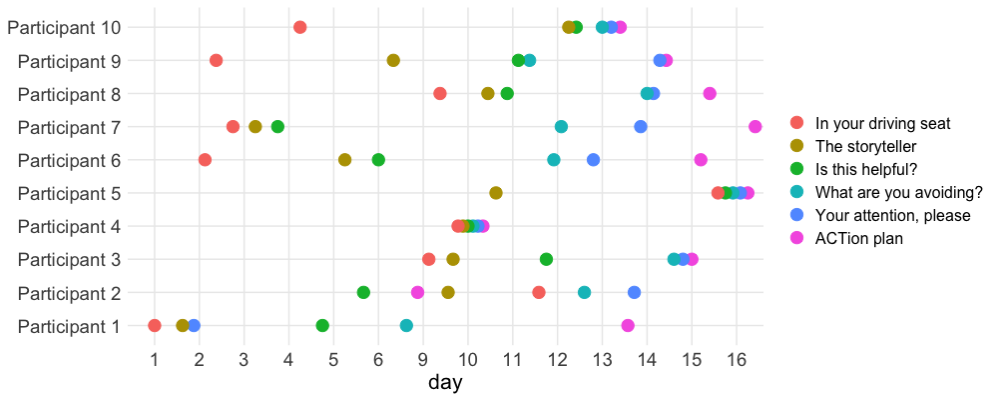
Completion order of ACT-based modules by participant. Participants onboarded onto the app over 2 days and had access to the app for 14 days.

All participants onboarded on to the app over 2 days and engaged with the app on average 5.8 minutes per day (median 6.0, IQR 4.9-7.4 minutes). The instructions to participants were to complete all 6 modules within 2 weeks, and there was considerable variability in the spacing between module completion (Figure 2); some participants completing 1 or 2 modules per day (n=6), some completed up to 3 in 1 day (n=2), and some completed all 6 modules in 1 day (n=2).

The most-used tool was the mood declaration (EMA); every participant completed at least seven declarations over the study (mean 13.1, median 13, IQR 9.3-14). As much as 9/10 participants (90%) also completed the deep breathing exercise (mean 2.4, median 2, IQR 1.3-2.8) and 6/10 participants (60%) completed the body scan exercise (mean 1.4, median 1, IQR 0-1.8).

### Usability

Results from the SUS indicate an average A rating (mean 83.4, median 88.7, IQR 75-96.9). All 7 participants that had an exit interview stated downloading the app and going through the app onboarding process including pairing the wearable were simple and none reported problems. Three participants stated they felt they needed to wear the wearable a bit too tight or that it was uncomfortable at times.

### Acceptability

All 7 participants that engaged in exit interviews stated that the ACT modules were relevant to the anxiety they currently struggled with. Two participants stated they had engaged in therapy before and, therefore, the ACT modules were not new concepts. All others stated the modules were relevant to, helpful, and useful for their anxiety. One participant noted the ease of use with each module being “quite short and didn’t take long to complete.” Two participants stated addressing overthinking and challenging thoughts (“The storyteller” and “Is this helpful?”) were particularly useful. Another 2 participants mentioned “What are you avoiding?” as the most useful module. One participant preferred the journaling activities on paper, rather than in the app.

Participants stated the tools were also relevant, useful, and helpful to their anxiety. Three participants stated the deep breathing exercise was therapeutic, especially before bed. Two participants specifically found the mood tracker helpful for their anxiety, stating “it made me stop and think a bit more about how I’m feeling.” One participant mentioned the body scan as the most useful tool as she preferred body awareness mindfulness techniques over breathing-related relaxation exercises.

Overall, 2 participants stated that by “using an app every day to check in with myself, it’s much easier to control my anxiety” and that “having goals set by the app and a module to complete each day was helping my mental state ... makes me look at [a feeling], acknowledge it and move on from it easier.”

### Preliminary Efficacy

#### Outcome Measures

Table 3 shows the descriptive statistics for baseline and exit for scores on psychological flexibility (AAQ-II), mental well-being (WEMWBS), and symptoms of anxiety (GAD-7) and depression (PHQ-9).

**Figure 3:**
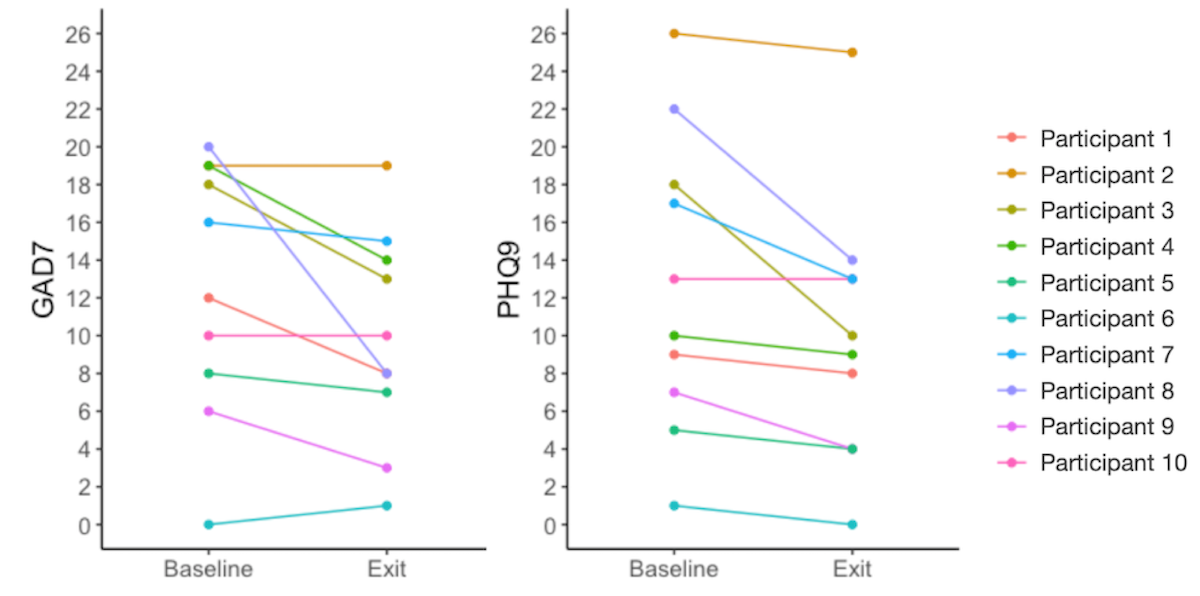
GAD-7 and PHQ-9 results in participants (N=10) of the digital intervention. The clinical cutoff for moderate anxiety or depression, respectively, is 10.

On average, participants had lower median scores for GAD-7 and PHQ-9 at exit than at baseline, indicating fewer anxious and depressive symptoms, respectively, and these differences were statistically significant (*P*=.03 and .008, respectively, for GAD-7 and PHQ-9; Table 3). For the GAD-7, the median exit score (9.8) fell below the threshold for moderate anxiety (score of 10) from a median baseline score of 12.8 (Figure 3).

Participants had lower median AAQ-II scores at exit than at baseline, indicating more psychological flexibility and better functioning, and higher median mental well-being scores, although this difference was not statistically significant (*P*=.11 and .55 for AAQ-II and WEMWBS, respectively; Table 3).

An additional analysis was conducted on 13 participants, which included 3 participants that did not complete all 6 modules, to determine if excluding these participants had a significant impact on the results. However, these results remain significant for change in GAD-7 (n=13; baseline median 12, exit median 8, Wilcoxon W=53, *P*=.011, *d*=0.81) and PHQ-9 (n=13; baseline median 10, exit median 8, Wilcoxon W=45, *P*=.008, *d*=0.84).

**Table 3:** Preliminary efficacy in participants (N=10) of the digital intervention. The clinical cutoff for GAD-7 and PHQ-9 for moderate anxiety or depression, respectively, is 10.

Measure	Baseline	Exit	Statistical significance
AAQ-II^a^, median (range)	30.5 (23.0-48.0)	29.0 (17.0-47.0)	W^e^=30; *P*=.11; ES^f^=0.512
WEMWBS^b^, median (range)	36.5 (17.0-58.0)	38.0 (22.0-56.0)	W=41.5; *P*=.55; ES=0.630
GAD-7^c^, median (range)	14.0 (0-20)	9.0 (1-19)	W=34.0; *P*=.03^g^; ES=0.691
PHQ-9^d^, median (range)	11.5 (1.0-26.0)	9.5 (0-25.0)	W=45; *P*=.008^h^; ES=0.840

^a^AAQ-II: Acceptance and Action Questionnaire-II.

^b^WEMWBS: Warwick-Edinburgh Mental Well-being Scale.

^c^GAD-7: 7-item Generalized Anxiety Disorder Assessment.

^d^PHQ-9: 9-item Patient Health Questionnaire.

^e^W: paired-sample Wilcoxon signed-rank tests.

^f^ES: effect size.

^g^*P*<.05.

^h^*P*<.01.

#### Interviews

Using the exit questionnaires, participants reported that the primary learned concept was that mental health consists of a number of factors; 3 participants specifically mentioned learning how much their sleep patterns play an important role. This was investigated further in interviews with participants.

Two participants mentioned they learned the most from the “Is this helpful?” module; 1 participant said, “how to divide your thoughts into those that are helpful and not helpful ... a way I haven’t looked at it before… even now, since I’ve stopped using it, if I have a thought, I think ‘what have you just gained from that thought?’ and if it’s not a productive thought, I need to either eradicate it or turn it into one. And I just found that really useful for a positive mindset”.

In terms of behavior change after the intervention, participants mentioned being more aware of their sleep patterns, consistently doing deep breathing and body scan exercises (without the guide on the app), checking in more with how they are feeling, and trying to make their goals more specific to avoid being overwhelmed.

## Discussion

### Principal Findings

This pilot study aimed to describe the development of a self-guided ACT-based digital prototype for individuals with GAD and to provide a feasibility analysis (ie, evaluate the acceptability, usability and preliminary efficacy) of the digital intervention. The study found that the content was acceptable to this population, and highly regarded due to its relevance for the participants’ struggle with anxiety. The intervention was judged to be usable in a digital format, with an “A” rating on the SUS scale. Additionally, preliminary evidence suggests that this intervention may reduce symptoms of anxiety and depression.

### Acceptability

Participants stated that they found the app and ACT-based content useful, easy to complete, and relevant to the anxiety they struggle with. Modules from all 3 pillars of ACT (Aware, Open, Active) were also mentioned as being useful. The therapeutic tools for mood tracking, deep breathing, and mindfulness alongside the novel ACT content were also mentioned as being helpful for managing symptoms. Additionally, using the app daily made it easier for participants to control their anxiety, acknowledge their mental state, and take actions that move them closer to achieving their goals. It is important to note the participants had been diagnosed with GAD and were not treatment naïve; 2 participants did not find the concepts novel. However, being reminded of these concepts was found to be helpful, thereby demonstrating the utility of the intervention.

### Usability

This study found the app-based therapeutic to have an SUS rating of 83.5, which equates to an A rating, representing programs that people are likely to recommend to their friends [68]. This is comparable to web-based ACT programs [27,29,62]. Three participants commented that the wrist-worn wearable was uncomfortable at times, but this did not affect their judgment of the intervention as simple and easy to use.

### Engagement

Of the 13 initial participants, 10 (77%) were included in the final analysis (3 participants did not complete all modules). This was comparable to another self-guided study using a 9-module, 2-week online program (75%; [27]) with similar financial incentives for the participants to take part, and on the higher end of other guided internet-based treatment programs (average adherence rates of 31%, range of 2%-83%) [37]. The dropout rate of this self-guided digital intervention (23%) is slightly higher than those of face-to-face, therapist-led ACT programs (15.8% [71] and 17.35% [72]).

Adherences rates in self-guided app-based ACT interventions are not consistently reported, therefore putting engagement results within the context of the wider literature is difficult. Across internet-based ACT treatments, 1 review found the average attrition rate was 19.2% [23], but made no separate analysis between guided (n=13) and unguided (n=5) interventions. Where higher dropout rates are observed [30,32], these could be explained by a lack of technical knowledge, lower motivation to engage with treatment, and poor usability of the system [25]. While this study found 65% (13/20) uptake rate, encouragingly, this pilot study indicates system usability was high and the adherence rate was on the higher end of the expected range.

### Analysis of Initial Efficacy

Although only a pilot acceptability study, the results provide preliminary evidence that this app-based therapeutic may be efficacious in reducing clinical symptoms in GAD. In this small sample, the results show trends of increasing psychological flexibility and well-being and decreasing symptoms of anxiety and depression. The large effect sizes found in this study are comparable to similar studies investigating digital ACT-based interventions on anxiety and depression [27,29,73], but due to the low sample size these results are in need of replication with larger sample sizes.

Symptoms of anxiety and depression were significantly decreased after 2 weeks (*P*=.03 and .008 for GAD-7 and PHQ-9, respectively). Mean GAD-7 scores decreased by 3 points (median 5 points) and mean PHQ-9 scores decreased by 2.8 points (median 2 points), but this may not represent a clinically significant change, as participants on average are at the cusp of the cutoff of 10, indicating moderate symptoms and a recommendation of a referral to a mental health professional. However, the reduction in anxiety and depression scores allude to the transdiagnostic effect of the treatment on comorbid symptoms [74] and highlights that a therapeutic solution for GAD should address the high comorbidity of anxiety and depression.

This study did not find a statistically significant increase in psychological flexibility (AAQ-II, *P*=.11), which is the primary objective of ACT-based interventions; however, given the small sample size, it is likely the study was underpowered to detect this effect. Future research with an increased sample size could investigate the mediating effect of psychological flexibility on anxiety and depression scores.

### Limitations

The feasibility phase of this study was limited by its small sample size and relatively short intervention period, and so conclusions are made with caution. Even though GAD is more common in females [3], the study was limited by a sample composed only of females. Future research should include measures of ethnicity [75] and aim for a sample more representative of the population with GAD. Compared with other PBA studies [44,76,77], the small sample size used in this study to represent the target population means that study may not have covered all context-specific behaviors that users with GAD may experience. In addition, the intervention was a prototype (6 modules) of a larger intervention (30 modules), and therefore only investigates the initial feasibility of the intervention concept. The intervention also comprises features that are known to decrease symptoms of anxiety, and these effects cannot be interpreted separately. Further research with a larger feasibility study on the 30-module intervention, that is more generalizable to a wider population of individuals with GAD, is warranted.

In addition, participants were paid to take part, meaning that engagement rates with the ACT modules in uncompensated participants are unknown. In a previous study using BioBase content related to workplace stressors, paid participants showed higher engagement in the program. However, there were no differences seen in the effect on outcome measures of well-being and anxiety. Future research on engagement should aim to use a similar incentive scheme to particular usage cases.

Although the prototype design process listed desired outcome behaviors of the intervention, the approach lacked clearly stated behavior change techniques for each of the desired behaviors [78]. Future iterations of the program will need to address this to increase engagement and efficacy of the intervention. In addition, barriers to use need to be further investigated, including the restrictions of using a technology-based solution as a therapeutic. For example, individuals with GAD may find technology useful to help soothe anxiety symptoms; however, technology can also be a tool to provide unhelpful distraction from difficult thoughts and feelings. In addition, technology can be used to connect with supportive social networks; however, feelings of being overwhelmed due to social media use can also increase symptoms of anxiety.

### Conclusions

The PBA approach is designed to complement and enrich the evidence-based approach to intervention design. This paper shows that the design and development of a persona and incorporation of intervention design objectives and key features alongside the app of an ACT-based model can be a feasible solution within in an app-based therapeutic. However, in keeping with the PBA approach, more research is needed to further define the engagement criteria for an efficacious product and further develop and tailor the ACT intervention to the target GAD population in keeping with the iterative development–evaluation–development cycles of the PBA.

## Appendix

Multimedia Appendix 1 Screenshots of the app.

## Abbreviations

AAQ-II: Acceptance and Action Questionnaire-II
ACT: acceptance and commitment therapy
DSM-5: Diagnostic and Statistical Manual of Mental Disorders, 5th edition
EMA: Ecological Momentary Assessment
GAD: generalized anxiety disorder
GAD-7: 7-item Generalized Anxiety Disorder Assessment
NICE: National Institute for Health and Care Excellence
PBA: person-based approach
PHQ-9: 9-item Patient Health Questionnaire
SUS: System Usability Scale
WEMWBS: Warwick-Edinburgh Mental Well-being Scale
