# Supplementary material for: Development and Feasibility of a Digital Acceptance and Commitment Therapy–Based Intervention for Generalized Anxiety Disorder: Pilot Acceptability Study
Source: JMIR Form Res. 2021 Feb 9;5(2):e21737. doi: 10.2196/21737 (PMC7902195; doi:10.2196/21737)
Supplement: Multimedia Appendix 1 [file formative_v5i2e21737_app1.pdf]

Mood tracker

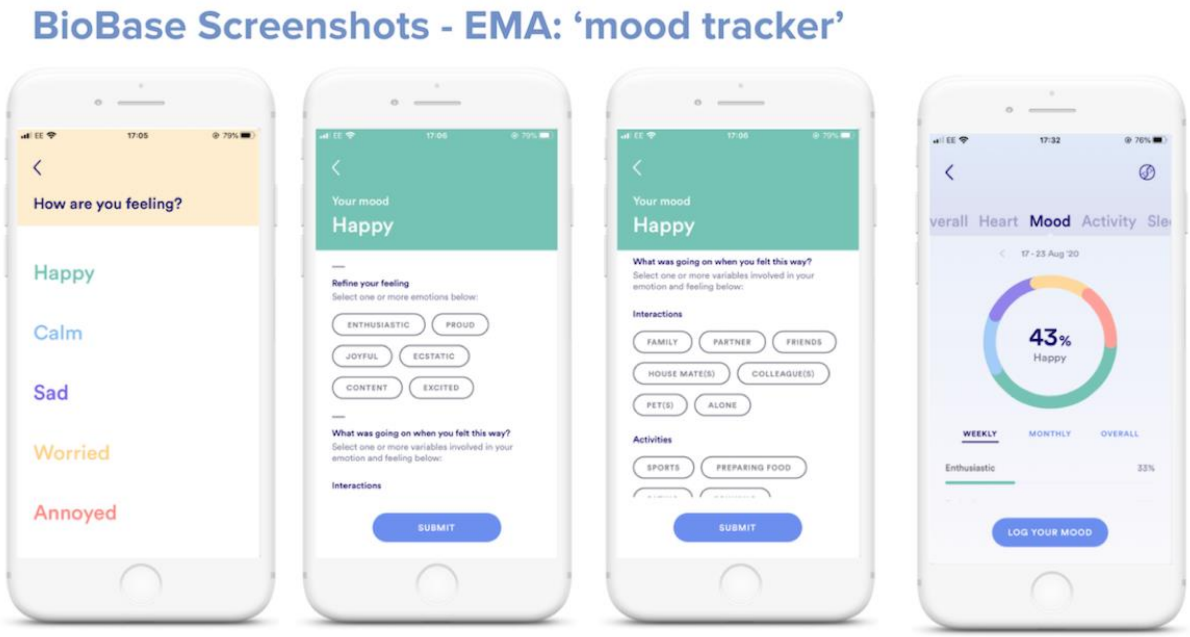

Tools and dashboards

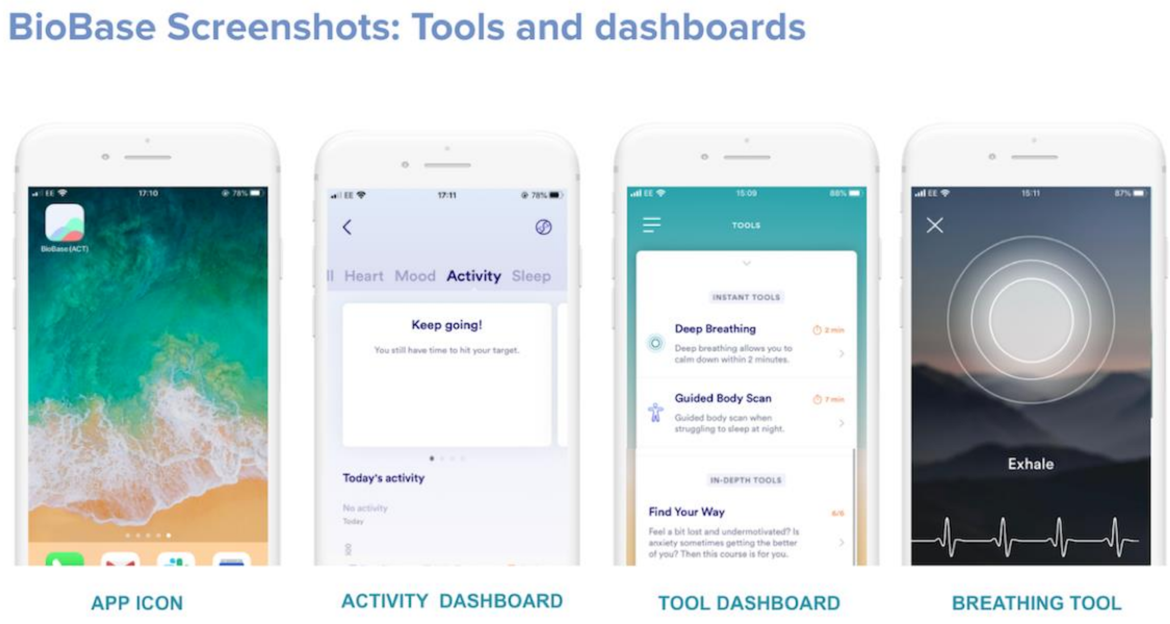

Modules

BioBase Screenshots: Modules

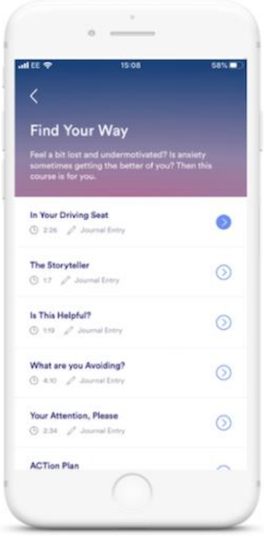

MODULE OPTIONS

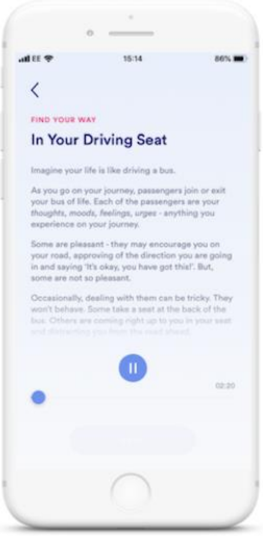

AUDIO OR TEXT

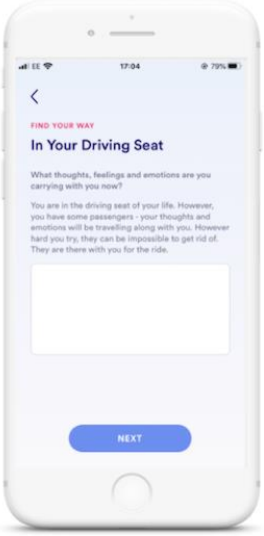

ACTIVITY WITH FREE TEXT

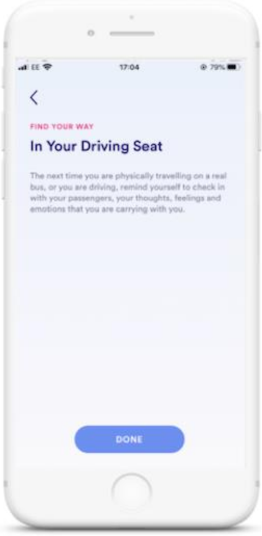

MODULE REVIEW
